# Vitamin D improves the antidiabetic effectiveness of aerobic training via modulation of Akt, PEPCK, and G6Pase expression

**DOI:** 10.1186/s13098-023-01158-y

**Published:** 2023-09-09

**Authors:** Zahra Hoseini, Nasser Behpour, Rastegar Hoseini

**Affiliations:** Department of Exercise Physiology, Faculty of Sport Sciences, Razi University, P.O. Box. 6714967346, Kermanshah, Iran

**Keywords:** Vitamin D, Exercise, Insulin resistance, Akt, PEPCK, G6Pas

## Abstract

**Background:**

Although the effect of Vitamin D Supplementation (Vit D) on several chronic diseases has been well conceded, its role in diabetes remains ambiguous. The present study investigated the interactive effects of Aerobic Training (AT) and different Vit D doses on Protein Kinase B (Akt), Phosphoenolpyruvate Carboxylase (PEPCK), and Glucose-6-Phosphatase (G6Pase) protein expressions in hepatocytes of type-2 diabetic rats.

**Methods:**

Fifty-six male Wistar rats were divided into 2 groups SHAM (non-diabetic control; n = 8), and diabetic (n = 48). Then, diabetic rats were divided into six groups: AT with high doses of Vit D (D + AT + HD), AT with moderate doses of Vit D (D + AT + MD), high doses of Vit D (D + HD), moderate doses of Vit D (D + MD), AT receiving vehicle (sesame oil; D + AT + oil), and control (oil-receiving). D + AT + HD and D + HD groups received 10,000 IU of Vit D; while D + AT + MD and D + MD groups receive 5000 IU of Vit D once a week by injection; D + AT + oil and SHAM groups received sesame oil. Diabetes was induced via intraperitoneal (IP) injection of streptozotocin (50 mg/kg body weight). After 2 months of intervention, serum insulin, glucose, and visceral fat were measured; protein expressions of Akt, PEPCK, and G6Pase were assessed by western blotting. The paired t-test, one-way analysis of variance (One-Way ANOVA), and the Tukey post hoc test were used at the signification level of P < 0.05.

**Results:**

Our data indicate that the diabeticization of rats increased the level of insulin, glucose, and PEPCK and G6Pase protein expressions and decreased the expression of the Akt (P < 0.05 for all variables). Combined AT and moderate or high Vit D significantly reduced body weight (P = 0.001; P = 0.001), body mass index (P = 0.001; P = 0.002), food intake (P = 0.001; P = 0.001) comparing the pre-test with the post-test, respectively. Also, AT and either high or moderate Vit D alone therapies lead to the improvement of the metabolic state, however, their combination had a more significant effect on the treatment of type 2 diabetes.

**Conclusions:**

Findings from the present study suggested that combined Vit D supplementation and AT successfully improve liver function and attenuate insulin resistance via upregulating Akt and downregulating PEPCK and G6Pase expressions, compared with monotherapy.

**Supplementary Information:**

The online version contains supplementary material available at 10.1186/s13098-023-01158-y.

## Introduction

Type 2 Diabetes mellitus (T2DM) is a chronic metabolic disease and the most common type of diabetes, being primarily identified by increased blood glucose, and insulin resistance [1], with decreased age of onset in recent years, up to 366 million individuals are expected to suffer from T2DM by 2030 [2]. Several lifestyle interventions are used for the management of blood glucose levels in T2DM [3]. Also, increased liver gluconeogenesis has been confirmed as a primary pathological phenomenon, often representing abnormal glucose metabolism in the liver [4].

Glucose-6-Phosphatase (G6Pase) and Phosphoenolpyruvate Carboxylase (PEPCK) are two fundamental liver enzymes converting non‑sugar substances into glucose [4, 5], and their abnormal expression is closely associated with enhanced gluconeogenesis, which is referred as a marker of T2DM [6]. Hepatocyte gluconeogenesis could be regulated under normal insulin signaling by both reducing glucose output and increasing peripheral tissue glucose uptake which is disturbed in T2DM [7, 8]. Therefore, hepatic insulin resistance is a substantial contributor to fasting hyperglycemia in type 2 diabetes [8]. Also, the increased circulating pancreatic insulin inhibits hepatic glucose output via activating the serine/threonine-Protein Kinase B (Akt) during feeding [9]. Based on these studies, PEPCK and G6Pase protein expressions regulate Akt activity [9, 10]. It has been recently reported that PEPCK and G6Pase protein expressions decrease insulin sensitivity via potentiating insulin-mediated suppression of the gluconeogenic program, through Akt phosphorylation [4].

Exercise Training is a major lifestyle therapy in the treatment of type 2 diabetes [11]. Both the acute and persistent effects of Aerobic Training (AT) on glucose disposal and uptake have crucial implications for T2DM patients in terms of transient regulation of glucose homeostasis and chronic metabolic control [12]. Increased activation and/or expression of fundamental proteins that regulate glucose metabolism might be among the molecular mechanisms associated with enhanced insulin following AT [13]. Many studies have demonstrated improved insulin signaling in response to AT in the hepatic tissue [14, 15]; however, the underlying exercise-mediated mechanisms for increased liver insulin sensitivity are not well studied.

Vitamin D (Vit D) supplementation is believed to be linked with gene polymorphism that impairs glucose metabolism [16, 17]. A recent study reported no contribution between extra Vit D supplementation and the prevention of diabetes [18], while some studies suggest that Vit D, as adjuvant therapy, can effectively improve insulin sensitivity and might serve anti‑gluconeogenesis properties in obesity-related diseases [17]. However, limited studies to date have investigated whether the protective mechanism of Vit D on diabetes is associated with hepatocyte gluconeogenesis and the regulation of gluconeogenesis‑related protein expression. Our recent study in elderly women with Vit D deficiency and NAFLD showed significantly reduced anthropometric indices, liver enzymes, and glycemic indices [19]. Hoseini et al. [20] investigated the interactive effect of AT and different doses of Vit D in female Wistar rats introducing AT with high-dose Vit D supplementation as a more favorable method for reducing weight and visceral fat in female Wistar rats with metabolic syndrome.

Therefore, finding interventions with anti‑insulin resistance and anti-gluconeogenic properties to target Akt, PEPCK, and G6Pase may be useful for the treatment of T2DM. Thus, we hypothesized that combination or monotherapy of AT and Vit D might be useful in improving glycemic indices, and the protein expression of Akt, PEPCK, and G6Pase in liver tissue of streptozotocin-induced diabetic rats.

## Materials

### Animal preparation

**Fig. 1:**
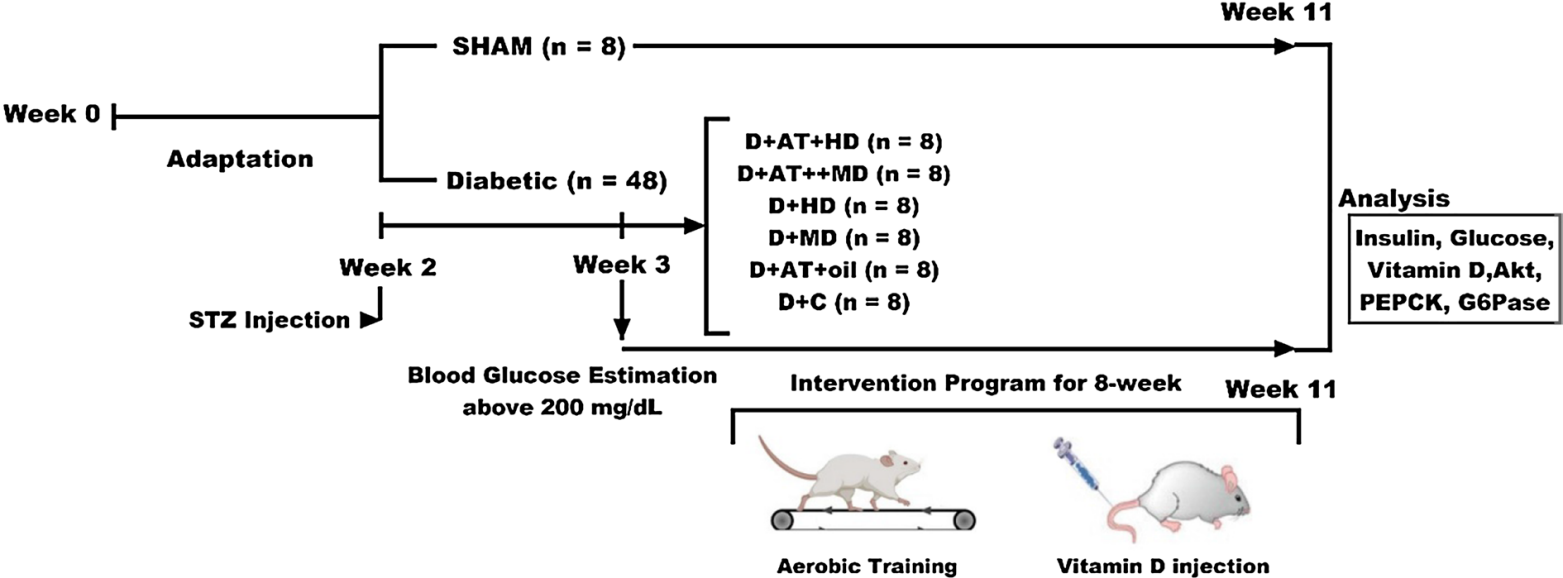
Flow chart of the study. D + AT + HD: diabetic + aerobic training + high dose of vitamin D; D + AT + MD: diabetic + aerobic training + moderate dose of vitamin D; HD: diabetic + high dose of vitamin D; MD: diabetic + moderate dose of vitamin D; D + AT + oil: diabetic + aerobic training + sesame oil; D + C: diabetic + sesame oil; SHAM: non-diabetic control

In this experimental study, fifty-six 10–12-week male Wistar rats were obtained from the Laboratory Animal Care Center of Medical Sciences University of Kermanshah and then were randomly assigned into two groups; non-diabetic healthy control (SHAM; n = 8) and diabetic (D; n = 48) (Fig. 1). The rats in the diabetic groups (n = 48) were then subdivided into six groups AT with high (D + AT + HD), and moderate (D + AT + MD) Vit D, high (D + HD), and moderate (D + MD) vitamin, AT receiving vehicle (sesame oil; D + AT + oil), and diabetic control (oil-receiving; D + C) groups. In order to determine the sample size, based on previous research, a moderate effect size of 0.5, a power level of 0.8, and a significance level of 0.05, with an estimated standard deviation of 1.5, and seven-group design with an equal allocation ratio was used suggesting a total sample size of 48 rats.

After 2 weeks of adaptation to the new environment, rats in the diabetic groups were fed High-Fat Diet in the form of pellets [HFD; a mixture of standard mouse food powder (365 mg/kg), yeast powder (1 mg/kg), mixed vitamins and minerals (60 mg/kg), sheep fat (310 mg/kg), chloride Sodium (1 mg/kg), and DL-methionine (3 mg/kg) (purchased from Beh-Parvar Company)] to induce obesity for 2 weeks [21, 22]. Diabetes was induced in rats by injecting nicotinamide solution (110 mg/kg body weight) and streptozotocin solution (50 mg/kg body weight) dissolved in citrate buffer (pH = 4.5) after weight increase (above 300 g). Diabetes (glucose levels above 200 mg/dL) was confirmed by measuring tail vein blood using a glucometer (Glucotrend 2, Roche Germany) after 2 weeks [23]. Animals received no insulin treatment throughout the study.

Rats were kept under a 12:12 h dark–light cycle, at a temperature of 21 ± 2 °C, and with a humidity level of 45–55%. They were housed in transparent polycarbonate shelves and provided with standard mouse food and water without any restrictions. All stages of the study were performed following the guidelines of the Ethics Committee of the Razi University of Kermanshah (IR.RAZI.REC.1401.065). All guidelines of laboratory animal studies were considered.

### Interventions

#### Vitamin D supplementation

The vitamin D receptor groups were divided into two protocols for the administration of vitamin D supplements (Table 1). The D + AT + HD and D + HD groups received a high dose of 10,000 IU/kg of vitamin D supplement weekly, while the D + AT + MD and D + MD groups received a moderate dose of 5000 IU/kg of vitamin D supplement weekly. It is worth noting that in both groups, almost 100 IU of vitamin D was derived from food sources. In addition to the oral supplementation, rats also received a weekly injection of vitamin D mixed with sesame oil. This injection served as an additional source of vitamin D, contributing to the overall intake of the participants. The utilization of sesame oil as a carrier for the injection ensured the stability and proper absorption of the vitamin D supplement [24].

**Table 1:** Numerical representation of the protocol in different weeks

Week	Acquaintance	1st	2nd	3rd	4th	5th	6th	7th	8th
Exercise duration (min)	5	15	15	20	20	25	25	30	30
Rolling speed (m/min)	10	10	10	15	15	20	20	25	25

#### Aerobic training

During the experimental sessions, each session began and ended with a 5-min warm-up and cool-down, respectively, at a moderate speed ranging from 5 to 10 m/min [25, 26]. The rats participated in aerobic training (AT) on a treadmill, 5 days a week over a duration of eight weeks. The training regimen commenced with a 15-min session at a speed of 10 m/min, gradually increasing to a 30-min session at a speed of 25 m/min, all performed on a treadmill set at a slope of 0 degrees. It is noteworthy that the running speed served as an indicator of the intensity of the aerobic training, with a range of 20 to 25 m/min considered as an average aerobic activity. This intensity level is estimated to be equivalent to approximately 70–75% of the maximum oxygen consumption [25, 27].

#### Body weight, body mass index, and food intake

Body weight (using a scale; Sartorius, Germany), Body length (using a tape measure; nose-to-anus), Body Mass Index (BMI), and food intake were recorded weakly between 8 and 10 AM. Food Intake (FI) was measured by subtracting the weight of the uneaten from the total given food [20].

#### Blood sampling

Forty-eight hours after the last training session, rats were anesthetized via intraperitoneal injection of xylazine (5 mg/kg) and ketamine (50 mg/kg) [28]. After ensuring full anesthesia, blood samples were taken by exposing the vena cava following the dissection of the abdominal cavity. The supernatant, designated as serum, was used to measure glucose, insulin, and serum 25(OH)D concentration after centrifuging the blood samples for 10 min at 4000 g. Serum insulin and glucose concentrations were determined using a rat insulin ELISA kit (DRG, Springfield Township, NJ, USA; intra-assay coefficient of variation = 1.62% and sensitivity of 1.76 mg/dL) and the colorimetric method (GOD-PAP, glucose oxidase aminoantipyrine; Pars Azmoun, Tehran, Iran), respectively. Homeostatic model assessment of insulin resistance (HOMA-IR) was calculated using the following equation [33]:

$$\left( {{\mathbf{fasting}}\;{\mathbf{insulin}}\left[ {{\mathbf{mU}}/{\mathbf{mL}}} \right) \times {\mathbf{fasting}}\;{\mathbf{glucose}}\left[ {{\mathbf{mmol}}/{\mathbf{L}}} \right]/{\mathbf{22}},{\mathbf{5}}} \right)$$

A rat 25(OH)D enzyme-linked immunosorbent assay (ELISA) kit (Immunodiagnostics system Ltd, Boldon, UK) was used to measure serum 25(OH)D concentration with an intraassay coefficient of variation and sensitivity of the method equal to 1.63% and 1.33 mg/dL, respectively.

#### Liver isolation, immunoprecipitation, and immunoblotting

The hepatic tissue was isolated, minced, and homogenized instantly in extraction buffer (containing 100 sodium fluoride, 100 sodium pyrophosphate, 10 sodium vanadate, 10 EDTA, 0.1 mg of aprotinin/mL, and 2 PMSF; pH 7.4, mM; 1% Triton-X 100, 100 Tris) using a Polytron PTA 20S generator (maximum speed, 48-degree Celsius, 30 s; model PT 10/35; Brinkman Instruments, Westbury, NY). First, the homogenized tissue was centrifuged for 40 min at 11,000 rpm and 48C to remove insoluble material. The tissue supernatants were used for immunoprecipitation (containing 2.0 mg total protein) with antibodies against Akt at 48C overnight, followed by SDS–PAGE, transferred to nitrocellulose membranes, and blotted with PEPCK, and G6Pase antibodies. The immunoblotting experiments were performed by separating the sample protein from150 mg liver protein extracts using SDS–PAGE, transferring to nitrocellulose membranes, and blotting with anti-Akt, anti-PEPCK, and anti-G6Pase (Santa Cruz Biotechnology, Inc., Santa Cruz, CA). Protein denaturation was done by boiling in Laemmli buffer in both immunoblotting [100 mM dithiothreitol (DTT)] and immunoprecipitation (50 mM DTT) experiments (Laemmli1970). A chemioluminescence kit was used to label specific bands (Sigma-Aldrich, St Louis, MO). Band intensities were visualized by X-ray film exposure of the membranes and quantified by optical densitometry (Scion Image Software; ScionCorp, Frederick, MD) [29].

### Statistical analysis

All data were expressed as mean ± SD. The Shapiro–Wilk was used to check the normality of data distribution, paired t-test analyzed the paired data to analyze paired data of body weight, BMI, and FI and one-way ANOVA followed by a Tukey test was used to compare between groups at significance level P < 0.05 using SPSS version 26.

## Results

Table 2 shows the alteration of body weight, BMI, FI, and in both diabetic rats (n = 48) and SHAM (n = 8). Significant differences were observed in body weight, BMI, FI, and between D and SHAM at the beginning and after 8 weeks of intervention. Body weight, BMI, and FI increased significantly in SHAM, while they decreased significantly in the D + AT + HD, D + AT + MD, D + AT + oil, D + HD, and D + MD groups at the end of the study compared with the beginning (P < 0.01) with the highest reduction in D + AT + HD (Table 2).

**Table 2:** Comparison of mean ± SD of body weight, BMI, FI and WC before and after intervention

Variables	D + AT + HD	D + AT + MD	D + HD	D + MD	D + AT + oil	D + C	SHAM	P-value^a^
Body weight (g)								
Before	316.87 ± 1.72	316.87 ± 1.72	308.75 ± 2.60	306 ± 2.50	313.87 ± 1.88	313.87 ± 1.88	224.12 ± 4.96	
After	281.62 ± 2.06	286.62 ± 2.38	293.37 ± 2.55	295.62 ± 2.26	293.50 ± 2.20	293.50 ± 2.20	228.87 ± 5.27	
P^†^	0.001*	0.001*	0.001*	0.001*	0.001*	0.001*	0.001*	
Δ	− 35.25 ± 0.462^A^	− 30.25 ± 2.434^B^	− 15.37 ± 0.517^D^	− 10.37 ± 0.744^E^	− 20.37 ± 0.517^C^	16.75 ± 0.464^G^	4.75 ± 0.460^F^	0.001^¥^
BMI (kg/m^2^)								
Before	0.78 ± 0.138	0.79 ± 0.061	0.83 ± 0.068	0.75 ± 0.083	0.81 ± 0.059	0.81 ± 0.105	0.60 ± 0.062	
After	0.70 ± 0.122	0.72 ± 0.056	0.79 ± 0.064	0.72 ± 0.081	0.76 ± 0.056	0.85 ± 0.111	0.61 ± 0.064	
P^†^	0.001*	0.002*	0.004*	0.011*	0.003*	0.004*	0.046*	
Δ	− 0.08 ± 0.0158^A^	− 0.06 ± 0.005^B^	− 0.04 ± 0.003^D^	− 0.02 ± 0.002^E^	− 0.05 ± 0.004^C^	0.04 ± 0.006^G^	0.01 ± 0.001^F^	0.001^¥^
FI (g/d)								
Before	20.25 ± 2.12	18.87 ± 1.35	17.25 ± 1.48	16.75 ± 1.48	18.12 ± 2.16	19.87 ± 1.88	12.75 ± 1.66	
After	16.37 ± 1.68	15.75 ± 1.28	15.12 ± 1.24	15.12 ± 1.52	15.43 ± 2.63	22.62 ± 1.76	13.37 ± 1.50	
P^†^	0.001*	0.001*	0.003*	0.013*	0.002*	0.001*	0.049*	
Δ	− 3.87 ± 1.246^A^	− 3.12 ± 0.640^ABCD^	− 2.12 ± 0.640^DE^	− 1.62 ± 0.231^E^	− 2.68 ± 0.883^CDE^	2.75 ± 0.462^β^	0.62 ± 0.744^F^	0.001^¥^

Data analysis was done by paired t-test, and the analysis of one-way analysis of variance test followed by post hoc Tukey’s test

BMI: body mass index; FI: food intake; D + AT + HD: diabetic + aerobic training + high dose of vitamin D; D + AT + MD: diabetic + aerobic training + moderate dose of vitamin D; HD: Diabetic + High Dose Of Vitamin D; MD: diabetic + moderate dose of vitamin D; D + AT + oil: diabetic + aerobic training + sesame oil; D + C: diabetic + sesame oil; SHAM: non-diabetic control

The mean values followed by different letters (A, B, C, D, E, F, and G) mean significantly different at the 0.05 level (p < 0.05). The values followed by the same letter are not significantly different. Dissimilar letters represent a significant difference between the groups

^†^P: Statistical analysis was done by paired sample t-test

*Significantly different in comparison pre and post-within the groups

^¥^Significantly different comparing Δ between groups

^a^P-value: Statistical analysis was done by one-way analysis test

As Table 3 shows, there was a significant difference in insulin, glucose, HOMA-IR, and serum 25-hydroxyvitamin D between the diabetic and SHAM groups. Furthermore, there were significant differences in insulin, glucose, and HOMA-IR between the diabetic groups, with the lowest level observed in the D + AT + HD group and the highest level observed in the D + C group. The results also indicate a statistically significant difference in serum 25-hydroxyvitamin D between the diabetic groups, except for the D + HD and D + MD groups (P = 0.189). The D + AT + HD group had the highest level of 25-hydroxyvitamin D, while the D + C group had the lowest level (Table 3).

Furthermore, the D + AT + HD, D + AT + MD, D + HD, D + MD, and D + AT + oil groups showed significantly higher levels of serum 25-hydroxyvitamin D compared to the D + C group (P < 0.05 for all three variables) (Table 3).

**Table 3:** Comparison of mean ± SD of visceral fat, insulin, glucose, HOMA-IR, and vitamin D after the intervention among the groups

Variables	D + AT + HD	D + AT + MD	D + HD	D + MD	D + AT + oil	D + C	SHAM	P-value^a^
Insulin (μU/mL)	3.33 ± 0.23^F^	3.51 ± 0.03^E^	4.52 ± 0.03^C^	4.90 ± 0.02^B^	3.81 ± 0.03^D^	6.21 ± 0.02^A^	1.66 ± 0.020^G^	0.001^¥^
Glucose (mmol/L)	173.75 ± 2.37^F^	214.25 ± 2.54^E^	240.62 ± 2.66^C^	266.37 ± 2.66^B^	233.37 ± 1.68^D^	292.87 ± 2.90^A^	121.75 ± 2.49^G^	0.001^¥^
HOMA-IR	1.43 ± 0.08^F^	1.86 ± 0.03^E^	2.68 ± 0.04^C^	3.22 ± 0.04^B^	2.19 ± 0.02^D^	4.49 ± 0.04^A^	0.49 ± 0.01^G^	0.001^¥^
Vit D (nmol/L)	143.75 ± 2.81^A^	125.62 ± 1.40^C^	140.62 ± 2.13^AB^	122.62 ± 2.32^CD^	86.37 ± 2.26^F^	80.12 ± 2.03^G^	104.62 ± 2.38^E^	0.001^¥^

Data analysis was done by the analysis of one-way analysis of variance test followed by post hoc Tukey’s test

HOMA-IR: homeostatic model assessment for insulin resistance; Vit D: serum 25-hydroxyvitamin D; D + AT + HD: diabetic + aerobic training + high dose of vitamin D; D + AT + MD: diabetic + aerobic training + moderate dose of vitamin D; HD: diabetic + high dose of vitamin D; MD: diabetic + moderate dose of vitamin D; D + AT + oil: diabetic + aerobic training + sesame oil; D + C: diabetic + sesame oil; SHAM: non-diabetic control

The mean values followed by different letters (A, B, C, D, E, F, and G) mean significantly different at the 0.05 level (p < 0.05). The values followed by the same letter are not significantly different. Dissimilar letters represent a significant difference between the groups

^¥^Significantly different between groups

^a^P-value: Statistical analysis was done by one-way analysis test

**Fig. 2:**
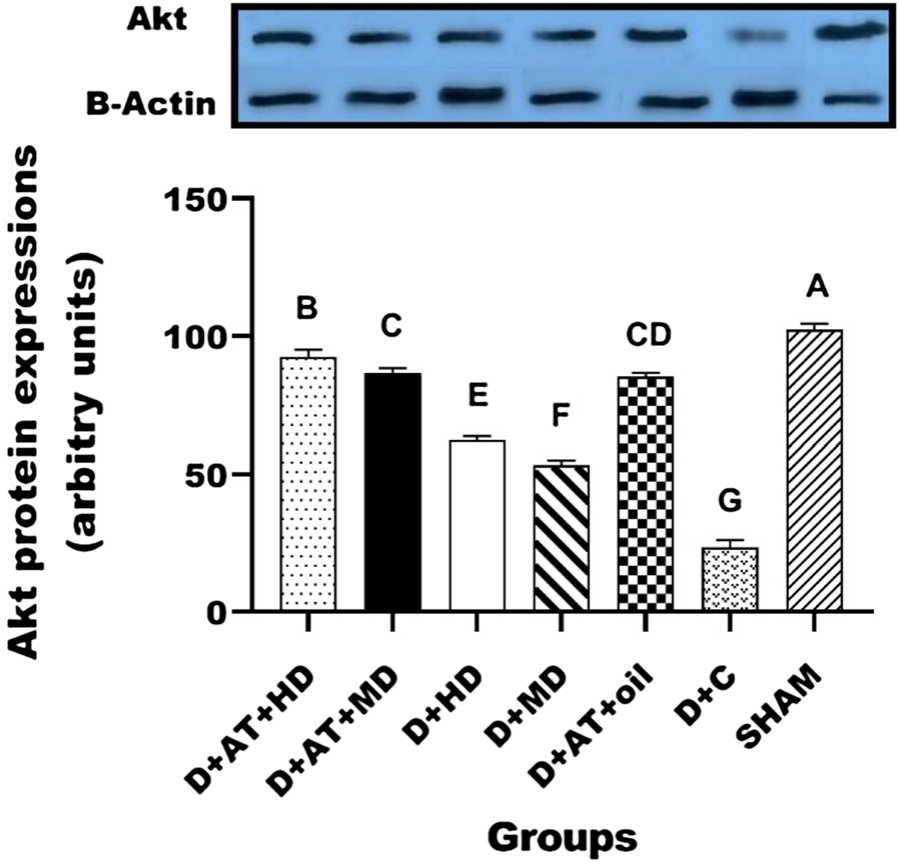
Comparison between mean ± SD of AKT protein expression between groups. D + AT + HD: diabetic + aerobic training + high dose of vitamin D; D + AT + MD: diabetic + aerobic training + moderate dose of vitamin D; HD: diabetic + high dose of vitamin D; MD: diabetic + moderate dose of vitamin D; D + AT + oil: diabetic + aerobic training + sesame oil; D + C: diabetic + sesame oil; SHAM: non-diabetic control. Values were calculated using a One-Way analysis of variance followed by post hoc Tukey’s test. The mean values followed by different letters (A, B, C, D, E, F, and G) mean significantly different at the 0.05 level (p < 0.05). The values followed by the same letter are not significantly different. Dissimilar letters represent a significant difference between the groups

**Fig. 3:**
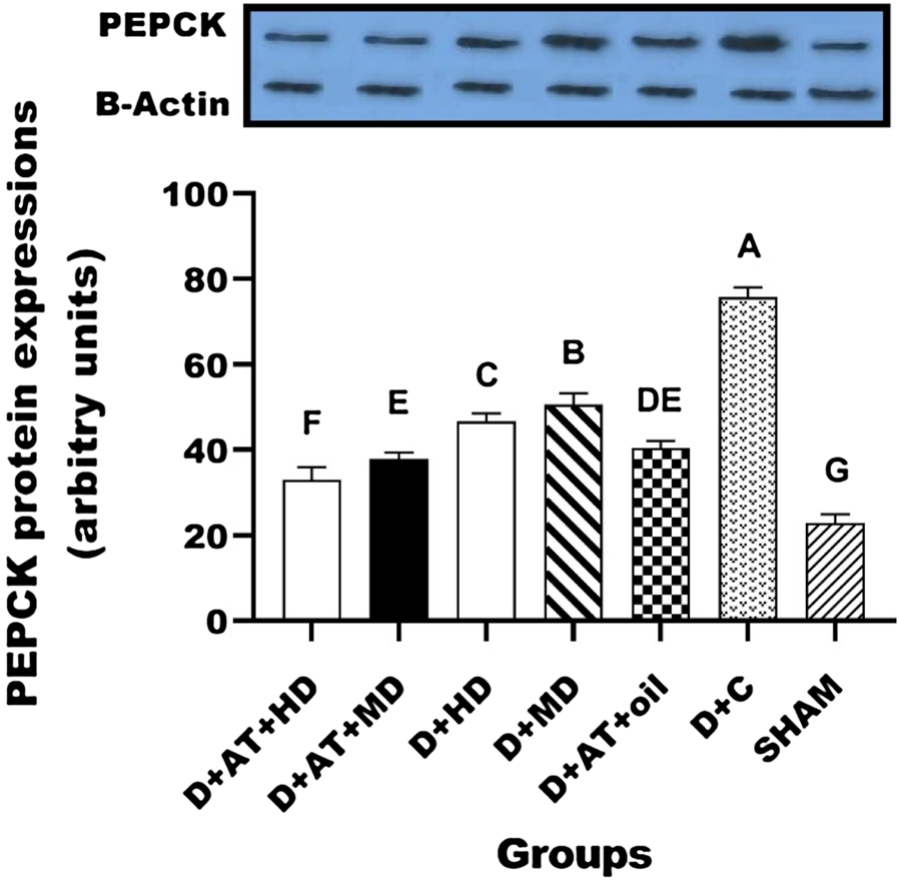
Comparison between mean ± SD of PEPCK protein expression between groups. D + AT + HD: diabetic + aerobic training + high dose of vitamin D; D + AT + MD: diabetic + aerobic training + moderate dose of vitamin D; HD: diabetic + high dose of vitamin D; MD: diabetic + moderate dose of vitamin D; D + AT + oil: diabetic + aerobic training + sesame oil; D + C: diabetic + sesame oil; SHAM: non-diabetic control. Values were calculated using a One-Way analysis of variance followed by post hoc Tukey’s test. The mean values followed by different letters (A, B, C, D, E, F, and G) mean significantly different at the 0.05 level (p < 0.05). The values followed by the same letter are not significantly different. Dissimilar letters represent a significant difference between the groups

**Fig. 4:**
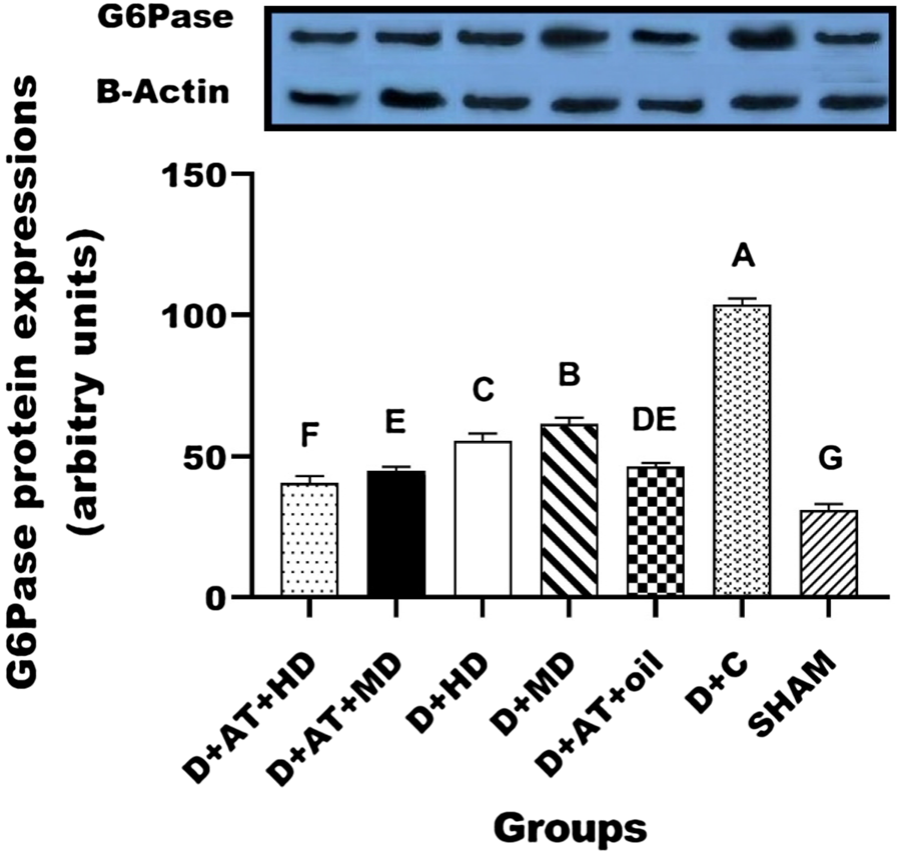
Comparison between mean ± SD of G6Pase protein expression between groups. **D** + AT + HD: diabetic + aerobic training + high dose of vitamin D; D + AT + MD: diabetic + aerobic training + moderate dose of vitamin D; HD: diabetic + high dose of vitamin D; MD: diabetic + moderate dose of vitamin D; D + AT + oil: diabetic + aerobic training + sesame oil; D + C: diabetic + sesame oil; SHAM: non-diabetic control. Values were calculated using a One-Way analysis of variance followed by post hoc Tukey’s test. The mean values followed by different letters (A, B, C, D, E, F, and G) mean significantly different at the 0.05 level (p < 0.05). The values followed by the same letter are not significantly different. Dissimilar letters represent a significant difference between the groups

The results of protein expression of Akt, PEPCK, and G6Pase are shown in Figs. 2, 3, and 4. The protein expression results for Akt, PEPCK, and G6Pase are depicted in Figs. 2, 3, and 4. There were significant differences observed in the protein expression of Akt, PEPCK, and G6Pase between the diabetic groups and the SHAM group. One-way ANOVA analysis also revealed significant differences in the protein expression of Akt, PEPCK, and G6Pase among the diabetic groups. Furthermore, the groups D + AT + HD, D + AT + MD, D + HD, D + MD, and D + AT + oil showed an upregulation of Akt and a downregulation of PEPCK and G6Pase compared to the D + C group (Figs. 2, 3, 4).

Based on the results, D + AT + HD exhibited a more significant upregulation of Akt (p = 0.024, p = 0.016, p = 0.001, and p = 0.001) and a downregulation of PEPCK (p = 0.029, p = 0.021, p = 0.001, and p = 0.001) and G6Pase (p = 0.019, p = 0.015, p = 0.002, and p = 0.001) compared to D + AT + MD, D + AT + oil, D + HD, and D + MD, respectively. However, the differences between D + AT + MD and D + AT + oil in terms of Akt upregulation and PEPCK and G6Pase downregulation were not statistically significant. Additionally, D + AT + MD induced a more significant upregulation of Akt (p = 0.001 and p = 0.001) and a downregulation of PEPCK (p = 0.001 and p = 0.001) and G6Pase (p = 0.002 and p = 0.001) compared to D + HD and D + MD, respectively.

Moreover, significant differences were observed in the protein expression of Akt, PEPCK, and G6Pase between D + HD and D + MD (P < 0.05 for all three variables).

## Discussion

This study investigated the effects of aerobic training and different doses of Vitamin D supplementation on body composition, glycemic control, and protein expression of key gluconeogenic proteins in streptozotocin (STZ)-induced diabetic rats. The results of this trial demonstrated the beneficial effects of an 8-week aerobic training (AT) with high or moderate doses of Vitamin D supplementation on improving body composition, fasting insulin (FI) levels, insulin metabolism, and the expression of Akt, PEPCK, and G6Pase in STZ-induced diabetic rats.

In line with previous studies [30, 31], all diabetic rats showed symptoms of type 2 diabetes mellitus (T2DM) such as weight gain and visceral fat accumulation due to hyperglycemia. However, this study found that 8 weeks of high or moderate Vitamin D supplementation, combined with AT, resulted in significant decreases in body weight, BMI, and FI in rats with T2DM. Importantly, these reductions were more significant when combining Vitamin D with AT compared to separate interventions. This suggests that adequate Vitamin D is crucial to achieving the beneficial effects of AT on weight loss. Consistent with our results, other studies have reported attenuated body weight, BMI, and FI following Vitamin D supplementation [32, 33]. Vitamin D supplementation may alter body weight by improving lipid metabolism through various mechanisms, such as inhibiting parathyroid hormone (PTH) secretion [34], altering lipoprotein lipase activity [35], suppressing the expression of uncoupling proteins in brown adipocytes [36], and affecting resting energy expenditure (REE) in adipose tissue [37], as well as decreasing fatty acid absorption [38].

Additionally, AT has been shown to increase the oxidation of adipose tissue [39, 40] and intramuscular triacylglycerols by increasing the catecholamine response [41, 42] and enhancing the ability of skeletal muscles to use fatty acids through increased adipose tissue and muscle blood flow [42, 43].

Our study found that 8-week 5000 IU/week Vitamin D supplementation significantly decreased insulin resistance in diabetic rats compared to the control (C) group. This finding is consistent with a study by Hoseini et al. [20], which reported that Vitamin D supplementation combined with AT significantly improves dyslipidemia and insulin resistance in ovariectomized rats. Another study by Hoseini et al. [20] suggested that 8-week exercise with 5000 IU/week Vitamin D supplementation reduced insulin levels and HOMA-IR significantly in Vitamin D-deficient women with fatty liver [19]. However, there are also studies with contradictory results [20]. For example, Garg et al. (2015) reported no significant effect on insulin secretion or resistance following 6 months of 4000 IU/day Vitamin D supplementation in Vitamin D-deficient women with polycystic ovary syndrome (PCOS) [44]. Insulin plays a critical role in regulating triglyceride and hepatic glucose production, stimulating glucose uptake in muscles, and inhibiting adipose tissue lipolysis and muscle proteolysis mechanisms. Insulin resistance can disrupt these processes, leading to metabolic complications [45, 46]. Vitamin D may regulate insulin metabolism by stimulating a second messenger system, leading to increased insulin secretion and sensitivity, possibly through increasing calcium influx and intracellular glucose within beta cells, as well as suppressing the release of pro-inflammatory cytokines [47]. Therefore, improved insulin metabolism by Vitamin D supplementation may decrease the risk of metabolic complications related to insulin resistance.

Based on the results of the present study, AT alone significantly decreased insulin resistance in diabetic rats compared to the high-dose (HD), moderate-dose (MD), and control (C) groups. Several molecular pathways could contribute to the decrease in insulin resistance following AT, including the upregulation of insulin-responsive transporters and insulin signal transduction [48, 49]. The activation of the insulin signaling pathway suppresses the gluconeogenic pathways [50], resulting in a rapid fall in hepatic glucose production. Furthermore, AT improves insulin sensitivity by reducing inflammatory cytokines and oxidative stress responses, thus ameliorating the pathophysiologic pathways of insulin resistance [51].

The major finding of this study was the upregulation of hepatic Akt expression, along with the downregulation of PEPCK and G6Pase expression, after the combination of Vitamin D supplementation, AT, and a high dose of Vitamin D compared to the control group. This was accompanied by a decrease in blood glucose concentration. Similar findings of enhanced hepatic insulin signaling and inhibited PEPCK activity have been reported in previous studies following physical exercise in animal models of obesity [52] or T2DM [53, 54].

In accordance with our results, earlier studies indicated an increase in liver Akt mRNA [55], and a decrease in PEPCK, and G6Pase following AT [54, 56]. However, the underlying exercise-mediated mechanisms need to be elucidated, Akt signaling pathway regulates the expression of CREB and FoxO1 which may be associated with the expression of PEPCK and G6Pase [4, 57].

Although, not much is known about the effects of simultaneous Vit D supplementation in mediating AT effects on hepatic metabolic regulators. The results of the present study represent a possibility of the interconnection between Akt, and gluconeogenic protein expressions (PEPCK and G6Pase) with Vit D receptor signaling pathways in the regulation of metabolism. These results suggest that gluconeogenic enzymes are associated with the improvement of hepatic insulin signaling and that exercise and Vit D supplementation are effective therapies for controlling diabetes by targeting hepatocyte gluconeogenesis regulation.

## Strengths and limitations

This study’s strength was using a randomized, placebo-controlled, single-blind trial with a low dropout rate, measuring and controlling daily food, and evaluating the protein expression alterations in liver tissue of streptozotocin-induced diabetic rats. There are a few limitations in this study. First, the evaluation of insulin resistance in the current study was only based on HOMA-IR. We did not evaluate the hepatic glucose production, and alterations of transcriptional factors such as TRB3, FoxO1, and PGC-1α signaling in the present study. Second, some of the insignificant results might be related to the short duration of the intervention. So, further studies are required with a longer duration and higher sample size to confirm our findings.

## Conclusions

Our data provide evidence that AT and Vit D supplementation improves insulin sensitivity by targeting hepatocyte gluconeogenesis and the regulation of gluconeogenesis‑related protein expression. Taken together, combined treatment with both AT and Vit D has an advantage in controlling diabetes via altering Akt, PEPCK, and G6Pase expression in animal models of T2DM. Further investigations are required to evaluate the effect of exercise on the selective Akt pathway inhibitors and the physiological relevance of this cross-talk.

### Supplementary Information

**Additional file 1.** Supplementary Figures.

## Data Availability

The datasets generated and analyzed during the current study are not publicly available due to ongoing data analysis but are available from the corresponding author upon reasonable request.

## Abbreviations

T2DM: Type 2 diabetes mellitus
Vit D: Vitamin D
AT: Aerobic training
Akt: Protein Kinase B
PEPCK: Phosphoenolpyruvate carboxylase
G6Pase: Glucose-6-phosphatase
UCP2: Uncoupling protein 2
REE: Resting energy expenditure
