# Supplementary figures and images for: Vitamin D improves the antidiabetic effectiveness of aerobic training via modulation of Akt, PEPCK, and G6Pase expression
Source: Diabetol Metab Syndr. 2023 Sep 9;15:184. doi: 10.1186/s13098-023-01158-y (PMC10492382; doi:10.1186/s13098-023-01158-y)

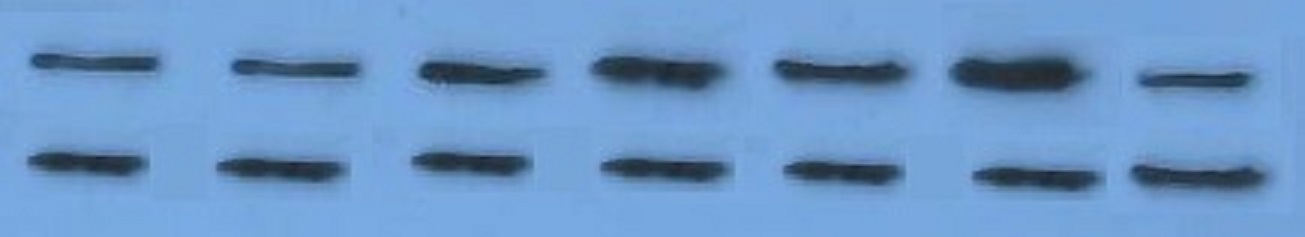

Supplement: Supplementary file 1 — Additional file 1. Supplementary Figures. [file 13098_2023_1158_MOESM1_ESM.zip › PEPCK.jpg]

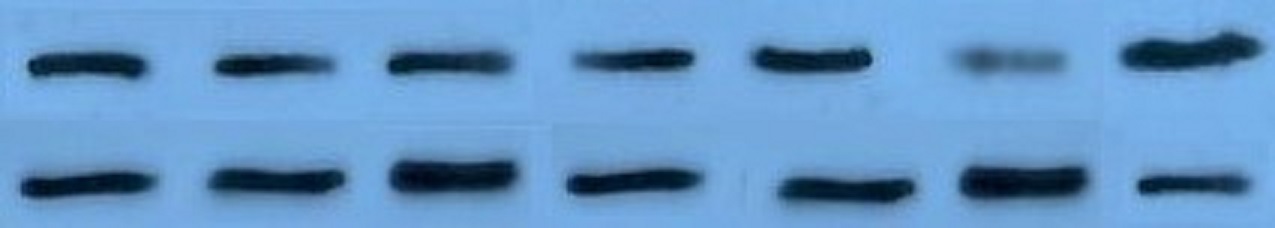

Supplement: Supplementary file 1 — Additional file 1. Supplementary Figures. [file 13098_2023_1158_MOESM1_ESM.zip › AKT.jpg]

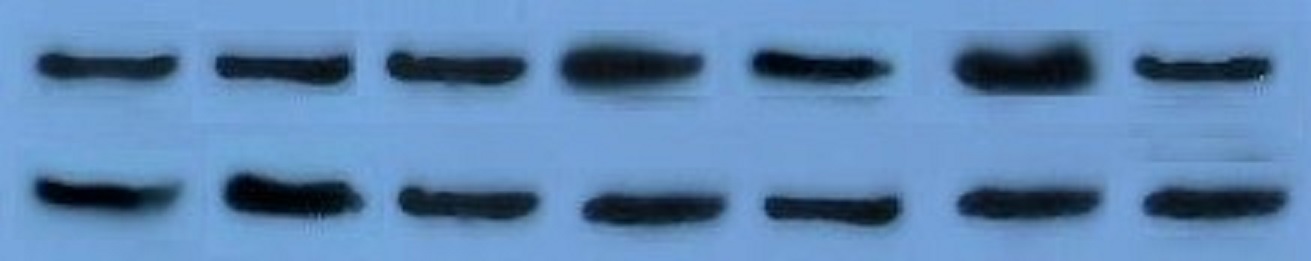

Supplement: Supplementary file 1 — Additional file 1. Supplementary Figures. [file 13098_2023_1158_MOESM1_ESM.zip › G6pase.jpg]
